# Expression of Serum Exosomal MicroRNA-21 in Human Hepatocellular Carcinoma

**DOI:** 10.1155/2014/864894

**Published:** 2014-05-18

**Authors:** Hongwei Wang, Lijuan Hou, Aihui Li, Yuxiu Duan, Haili Gao, Xinwen Song

**Affiliations:** Department of Infection Diseases, The First Affiliated Hospital of Xinxiang Medical University, Weihui 453100, China

## Abstract

New strategies for the diagnosis of hepatocellular carcinoma (HCC) are urgently needed. There is an increasing interest in using microRNAs (miRNAs) as biomarkers in diseases. In this study, we examined the expression of miR-21 in serum exosomes from patients with HCC or chronic hepatitis B (CHB) and investigated the potential clinical significance of miR-21. Quantitative RT-PCR indicated that the concentration of miR-21 was significantly higher in exosomes than in exosome-depleted supernatants or the whole serum. Further, the expression level of serum exosomal miR-21 was significantly higher in patients with HCC than those with CHB or healthy volunteers (*P* < 0.0001, *P* < 0.0001, resp.). High level of miR-21 expression correlated with cirrhosis (*P* = 0.024) and advanced tumor stage (*P* = 0.001). Although serum level of miR-21 was higher in patients with HCC than in patients with CHB and healthy volunteers, the sensitivity of detection is much lower than using exosomal miR-21. These findings indicate that miR-21 is enriched in serum exosomes which provides increased sensitivity of detection than whole serum. Exosomal miR-21 may serve as a potential biomarker for HCC diagnosis.

## 1. Introduction

Hepatocellular carcinoma (HCC) is the leading cause of cancer-related deaths in many Asian countries and the third most frequent cause of cancer deaths worldwide [1, 2]. Although surgical resection may be a curative treatment for HCC, many patients are nonoperable due to the lack of effective tools for early detection and diagnosis. This fact results in the high mortality rate. Therefore, the identification of sensitive and specific biomarkers for early detection of HCC is desirable and urgently needed.

MicroRNAs (miRNAs), ranging from 19 to 25 nucleotides in length, are frequently dysregulated in cancer and associated with cancer development and progression [3, 4]. MiRNAs are ideal candidates for biomarkers because of their resistance to endogenous RNase and high stability under different storage conditions [5]. Recent studies have shown that human serum miRNAs are aberrantly expressed in many malignancies such as liver, colorectal, and pancreatic cancer [6–8]. Increasing evidence suggests that unique serum miRNA expression signatures may serve as new noninvasive biomarkers for cancer diagnosis including HCC.

Exosomes are small membranous vesicles (30–100 nm) that are secreted by most cell types and can be isolated from various body fluids such as serum, urine, and malignant ascites [9–11]. Exosomes contain unique miRNAs, mRNAs, and proteins which may reveal genetic information of their parent cells [12]. Moreover, it was reported that the majority of serum miRNAs are enriched in exosomes [13]. Thus, exosomal miRNAs can serve as valuable noninvasive biomarkers for the diagnosis and prognosis of diseases [14]. However, to our knowledge, the potential of using exosomal miRNAs as a source for biomarkers in HCC has not been reported.

Therefore, we investigated the expression of microRNA-21 (miR-21), a prominently expressed miRNA in many human cancers including HCC [15–18], in exosomes isolated from serum samples from healthy volunteers and HCC or CHB patients. Further, we evaluated the potential of using serum exosomal miR-21 for early detection of HCC. The results of our study shed new light on the identification of new diagnostic and prognostic markers for the deadly HCC.

## 2. Materials and Methods

### 2.1. Participants

Blood samples from 30 patients with HCC and 30 patients with active chronic hepatitis B (CHB) were obtained at the First Affiliated Hospital, Xinxiang Medical University (Xinxiang, China), prior to definitive therapy. The tumor type and the grade of cell differentiation were diagnosed based on the criteria of World Health Organization (WHO), whereas the pathological stage of each tumor was determined by the International Union Against Cancer (UICC) TNM classification. Blood samples were also collected from 30 healthy volunteers with matching ages and genders to the patients. Written consents were obtained from all subjects prior to the recruitment. The study protocol was approved by the Institutional Review Board of Hospital Ethics Committee. The clinical characteristics of the subjects are listed in Table 1.

### 2.2. Exosome Isolation from Serum Samples

Peripheral blood was collected and centrifuged at 3,000 rpm for 10 min at 4°C to spin down the blood cells. The supernatants were centrifuged at 12,000 g for 10 min at 4°C to completely remove cellular components. The serum samples were stored at −80°C until use. Exosomes were isolated from serum samples using Total Exosome Isolation Reagent (from serum) following the manufacturer's protocol (Invitrogen). Briefly, 0.2 mL of the Total Exosome Isolation Reagent was added to 1 mL of serum and incubated for 30 min at 4°C, followed by centrifugation at 10,000 ×g for 10 min at room temperature. The exosome pellets were collected for characterizations and RNA extractions.

### 2.3. Transmission Electron Microscopy (TEM)

The exosome pellets were resuspended in PBS and placed onto Formvar carbon coated electron microscopy grids (Electron Microscopy Sciences, Hatfield, PA). After incubation for 5 min at room temperature, exosomes were fixed in 2% paraformaldehyde and washed twice with water. The grids were then negatively stained with 10% uranyl acetate for 10 min. The preparations were examined and photographed with a JEOL 100XCII electron microscope (JEOL, Peabody, MA).

### 2.4. Western Blot Analysis

The exosome pellets were lysed with RIPA buffer supplemented with protease inhibitors (Roche Applied Science, Indianapolis, IN). Western blot analysis was performed with exosome lysates as previously described [13].

### 2.5. Quantification of Serum MiRNAs

Total RNA was isolated from the exosomal pellets, the exosome-depleted supernatant, and whole serum using isothiocyanate-phenol/chloroform extraction procedures. miR-21 expression was examined as previously described [13]. Real-time quantitative RT-PCR (qRT-PCR) was performed using SYBR Premix DimerEraser kit (TaKaRa, Shiga, Japan) on an ABI Prism 7900HT Detection System (Applied Biosystems, Foster City, CA). U6 snRNA was used as an internal control. The primers for miR-21 and U6 were purchased from RiboBio (Guangzhou, China).

### 2.6. Statistical Analysis

The Mann-Whitney test and Kruskal-Wallis test were performed to determine the significance of the differences in serum miR-21 levels. All statistical analyses were done with STATA 10.0 (StataCorp LP, College Station, TX). A *P* value of <0.05 was considered significant.

## 3. Results

### 3.1. Characterization of Isolated Serum Exosomes

**Figure 1:**
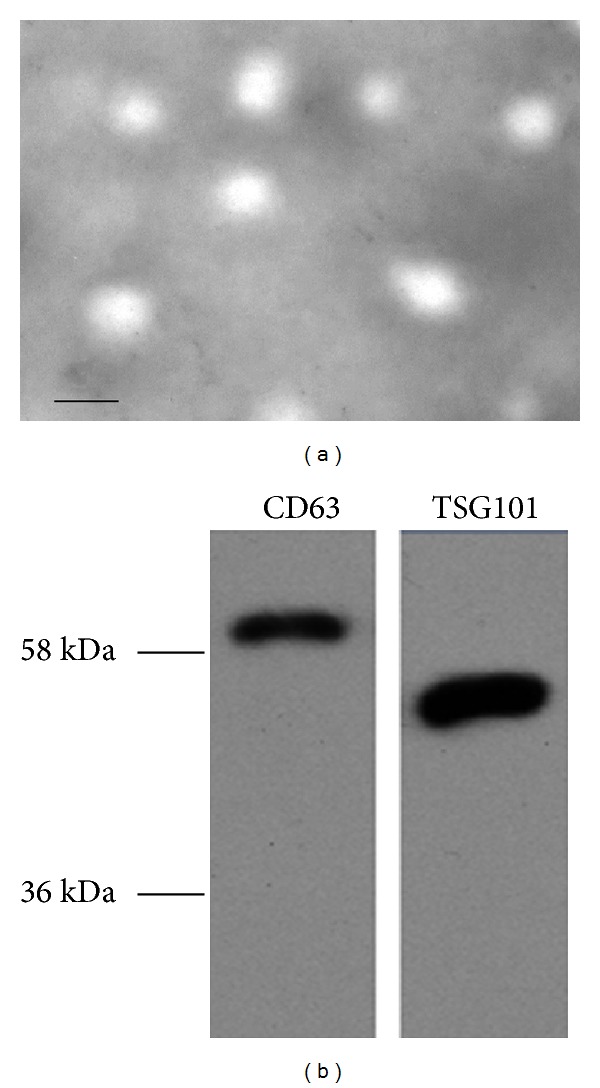
Validation of exosomes isolated from serum samples. (a) Morphological characterization of exosomes isolated from serum samples by transmission electron microscopy. Bar, 100 nm. (b) Expression of exosomal protein markers (CD63 and TSG101) in exosomes isolated from serum samples was assessed by Western blot analysis.

To ensure the efficacy and quality of the serum exosome isolation, we characterized the microvesicles by TEM and Western blot analysis. Electron microscopic analysis of the exosomes isolated from serum samples showed round structures with sizes varying between 50 and 100 nm (Figure 1(a)), consistent with previously reported characteristics of exosomes [19]. Further, the identification of exosomes was confirmed by the detection of specific exosomal protein markers CD63 and TSG101 using Western blot analysis (Figure 1(b)). These results confirmed successful isolation of exosomes from serum samples.

### 3.2. Serum Exosomal MiR-21 Expression Is Significantly Higher in HCC Patients

**Figure 2:**
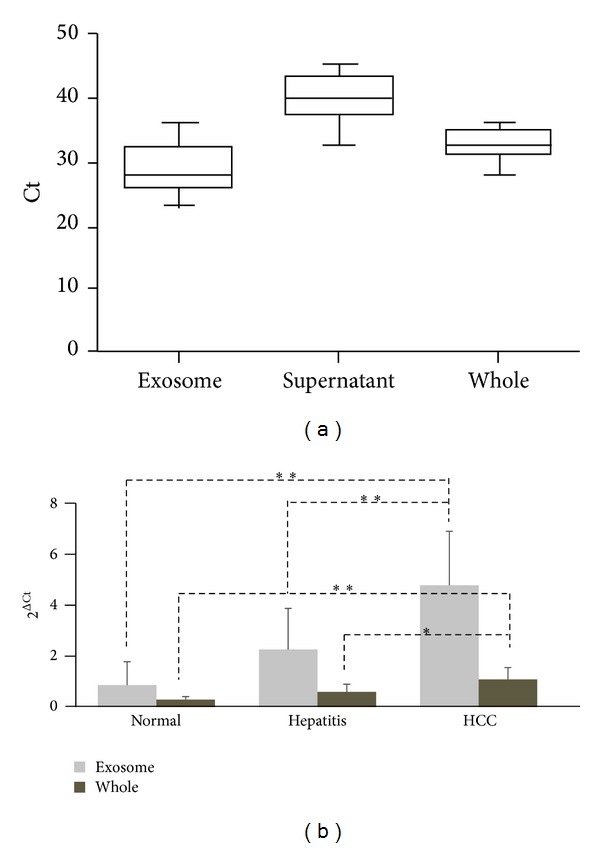
Serum exosomal miR-21 expression is significantly higher in HCC patients. (a) Serum miR-21 predominantly exists in exosomes. miR-21 levels in serum exosomes, exosome-depleted supernatants, and whole serum were determined by qRT-PCR. The absolute Ct values are presented. (b) The miR-21 levels in exosomes or whole serum samples from 30 patients with HCC, 30 patients with CHB, and 30 healthy controls were determined by qRT-PCR. *P < 0.001; **P < 0.0001.

MiRNA expression in exosomes isolated from serum samples of HCC patients has not been investigated. To determine if miRNAs in HCC serum are enclosed in exosomes and/or are circulating freely, we first extracted RNA from both exosome pellets isolated from 10 control serum samples and the exosome-depleted serum supernatants. MiR-21 expression was examined by qRT-PCR. Notably, miR-21 concentration was significantly higher in the exosomes than in the exosome-depleted supernatants (*P* < 0.01, Figure 2(a)). To examine miR-21 expression in whole serum, we extracted RNA directly from the 10 serum samples used above and quantified miR-21 expression. The concentration of miR-21 in the whole serum samples was lower than in the exosomes, but higher than in the exosome-free supernatants (Figure 2(a)).

Further, to investigate whether there is a potential benefit in using miRNAs in exosomes versus in the whole serum as biomarkers, we analyzed miR-21 levels in serum exosomes and whole serum in a cohort of 30 HCC patients, 30 CHB patients, and 30 healthy volunteers. The qRT-PCR results showed that exosomal miR-21 expression in patients with HCC was significantly higher than in CHB patients or healthy controls (2.12-fold, *P* < 0.0001 and 5.57-fold, *P* < 0.0001, resp.) (Figure 2(b)). The same results were observed in the whole serum samples, although to a lesser extent (1.62-fold, *P* < 0.001 and 3.91-fold, *P* < 0.0001, resp.) (Figure 2(b)). Importantly, miR-21 levels were significantly higher in exosomes than in the whole serum (*P* < 0.05). These observations indicate improved sensitivity of HCC detection using exosomal miR-21 (Figure 2(b)).

### 3.3. Serum Exosomal MiR-21 Correlates with the Clinicopathological Features of HCC

To better understand the potential roles of serum exosomal miR-21 in HCC development and progression, the Mann-Whitney test and Kruskal-Wallis test were performed to determine the potential association of serum exosomal miR-21 levels with various clinicopathological features of HCC. In this study, the average fold increase of miR-21 in HCC samples versus healthy control was 5, which was used as the cut-off value to divide the samples into high miR-21 expression (≥5-fold) and low miR-21 expression (<5-fold) groups. The statistical analysis revealed that high level of serum exosomal miR-21 expression positively correlated with cirrhosis (*P* = 0.024) and tumor stage (*P* = 0.001, Table 2). However, there was no correlation of miR-21 expression with other clinical features including age, gender, and HBV infection.

## 4. Discussion

HCC is one of the most common cancers worldwide with high mortality rate. Currently, the diagnosis of HCC relies on imaging characteristics obtained using computed tomography (CT) and/or magnetic resonance imaging (MRI). However, early detection and diagnosis of small lesions are relatively difficult and inaccurate [20]. Many cases are already nonoperable when diagnosed. Therefore, there is an urgent need for new and more sensitive biomarkers for early detection of HCC.

The finding that miRNAs can be detected in various biofluids including serum has opened up new opportunities in the search for biomarkers in cancer. High serum miRNA levels in cancer are considered to be due to excessive secretion by primary cancer cells. Exosomes are secreted by most cell types including cancer cells. They are found in all body fluids [21]. Serum exosomes are highly enriched in miRNAs. For instance, miR-21 was 40-fold higher in glioblastoma serum exosomes than in exosome-depleted fractions [9]. In prostate cancer, urine exosomal miR-107 and miR-574-3p are upregulated [22]. In this study, we showed that exosomes significantly improve the sensitivity of miRNA detection in serum. These observations indicate that exosomes may serve as very attractive means for noninvasive diagnosis [23].

Serum miR-21 was an independent significant factor for recurrence and was reported to be more sensitive than *α*-fetoprotein (AFP) for the detection of HCC [17]. Hence, we used miR-21 as an example to examine miRNA expression in whole serum and serum exosomes in HCC and CHB patients and healthy volunteers. We further evaluated the potential of using exosomal miRNAs clinically for early detection of HCC. We observed that miR-21 expression was significantly higher in exosomes than in exosome-depleted serum and the whole serum. Further, we showed that individuals with HCC had significantly elevated levels of serum exosomal miR-21, and the high level of miR-21 expression correlated with cirrhosis and tumor stage. This observation suggests that serum exosomal miRNAs, such as miR-21, may be useful for the prediction of HCC risk and early detection of HCC. Consistent with the results published by Tomimaru et al. [17], we found that serum miR-21 expression in HCC is higher than in CHB. However, another study reported the opposite result showing CHB patients express higher levels of serum miR-21 than HCC patients [24]. This discrepancy may be due to patient selection, miRNA detection methods, and the lack of common miRNA internal control.

In summary, we observed upregulated expression of exosomal miR-21 in serum obtained from HCC patients compared with in serum from CHB patients or healthy controls. Rapid advances in exosome isolation technology and the realization that exosomes contain valuable information on diseases and conditions have promoted the research on the utility of exosomes as potential diagnostic and prognostic tools [25]. Furthermore, screening a large cohort of serum samples from HCC patients would determine the value of differentially expressed exosomal miRNAs as potential biomarkers for early detection of liver cancer.

## Figures and Tables

**Table 1:** Clinical and pathological characteristics of patients and volunteers enrolled in the study.

	Healthy control	Chronic hepatitis B	HCC
Case, *n*	30	30	30
Age (years)	60.5 ± 7.8	62.0 ± 9.6	62.8 ± 8.1
Sex			
Female	9	9	7
Male	21	21	23
HBV status			
Positive		22	24
Negative	30	8	6
Liver cirrhosis			
Positive	0	0	23
Negative	30	30	7
TNM staging			
I			10
II			7
III-IV			13

**Table 2:** Correlations of patient clinicopathologic characteristics with exosomal miR-21 expression levels.

Characteristics	*n*	miR-21 expression		*P* value^1^
		Low *n* (%)	High *n* (%)	
Age (years)				
<60	11	7 (63.6)	4 (36.4)	1.000
≥60	19	11 (57.9)	8 (42.1)	
Gender				
Male	23	13 (56.5)	10 (43.5)	0.669
Female	7	5 (71.4)	2 (28.6)	
HBsAg				
Positive	24	13 (54.2)	11 (45.8)	0.358
Negative	6	5 (83.3)	1 (16.7)	
Liver cirrhosis				
Presence	23	11 (47.8)	12 (52.2)	0.024
Absence	7	7 (100.0)	0 (0.0)	
Tumor stage				
I	10	9 (90.0)	1 (10.0)	0.001
II	7	5 (71.4)	2 (28.6)	
III-IV	13	4 (30.8)	9 (69.2)	

^1^ *P* < 0.05.
